# Tissue Specific Induction of p62/*Sqstm1* by Farnesoid X Receptor

**DOI:** 10.1371/journal.pone.0043961

**Published:** 2012-08-27

**Authors:** Jessica A. Williams, Ann M. Thomas, Guodong Li, Bo Kong, Le Zhan, Yuka Inaba, Wen Xie, Wen-Xing Ding, Grace L. Guo

**Affiliations:** 1 Department of Pharmacology, Toxicology, and Therapeutics, University of Kansas Medical Center, Kansas City, Kansas, United States of America; 2 Department of Abdominal Surgery, Cancer Treatment Center, The Fourth Affiliated Hospital of Harbin Medical University, Harbin, People’s Republic of China; 3 Center for Pharmacogenetics, University of Pittsburgh, Pittsburgh, Pennsylvania, United States of America; Clermont Université, France

## Abstract

**Background:**

Farnesoid X Receptor (FXR) is a member of the nuclear receptor superfamily and is a ligand-activated transcription factor essential for maintaining liver and intestinal homeostasis. FXR is protective against carcinogenesis and inflammation in liver and intestine as demonstrated by the development of inflammation and tumors in the liver and intestine of FXR knock-out mice. However, mechanisms for the protective effects of FXR are not completely understood. This study reports a novel role of FXR in regulating expression of *Sqstm1,* which encodes for p62 protein. p62 plays an important role in maintaining cellular homeostasis through selective autophagy and activating signal transduction pathways, such as NF-κB to support cell survival and caspase-8 to initiate apoptosis. FXR regulation of *Sqstm1* may serve as a protective mechanism.

**Methods and Results:**

This study showed that FXR bound to the *Sqstm1* gene in both mouse livers and ileums as determined by chromatin immunoprecipitation. In addition, FXR activation enhanced transcriptional activation of *Sqstm1 in vitro*. However, wild-type mice treated with GW4064, a synthetic FXR ligand, showed that FXR activation induced mRNA and protein expression of *Sqstm1*/p62 in ileum, but not in liver. Interestingly, FXR-transgenic mice showed induced mRNA expression of *Sqstm1* in both liver and ileum compared to wild-type mice.

**Conclusions:**

Our current study has identified a novel role of FXR in regulating the expression of p62, a key factor in protein degradation and cell signaling. Regulation of p62 by FXR indicates tissue-specific and gene-dosage effects. Furthermore, FXR-mediated induction of p62 may implicate a protective mechanism of FXR.

## Introduction

Autophagy was strictly thought of as a bulk protein degradation pathway until the discovery that it also performs selective degradation of polyubiquitinated proteins via sequestosome-1(*Sqstm1*), which encodes for p62 protein. p62 is often found in cellular protein aggregates because it interacts with ubiquitinated proteins through its C-terminal ubiquitin associated (UBA) domain [1]. p62 also interacts with microtubule light chain 3 (LC3), an autophagy protein, via its LC3 interacting region (LIR). In addition to protein aggregates, recent studies indicate that p62 is also recruited to damaged mitochondria via binding to ubiquitinated outer mitochondrial membrane proteins, although this role of p62 in mitophagy is controversial [2], [3]. Therefore, p62 may serve as an autophagy receptor for ubiquitinated proteins and damaged mitochondria.

In addition to its role in autophagy, p62 also has a role in signal transduction and aids in a cell’s decision to undergo apoptosis or survival through its organization of signaling complexes in the cytoplasm [1], [4], [5]. Upon cytokine stimulation, p62 is able to activate the nuclear factor kappa-light chain-enhancer of activated B cells (NF-κB) pathway [5]–[7]. Activated NF-κB induces the expression of pro-survival genes, such as anti-apoptosis and cell proliferation genes. Activated NF-κB also induces the expression of inflammatory genes such as cytokines, chemokines, and adhesion molecules [8]. In addition, p62 activates nuclear factor erythroid 2-related factor 2 (Nrf2) by binding to kelch-like ECH-associated protein 1 (Keap1), which is important for inducing expression of genes involved in the oxidative stress response [9]–[11]. Finally, p62 is able to fully activate caspase-8 in the extrinsic apoptosis pathway, which results in the initiation of apoptosis and cell death [4]. Ultimately, p62 helps maintain cellular homeostasis through its participation in autophagy and signal transduction. Therefore, a defect in autophagy can cause an accumulation of damaged organelles and p62-bound protein aggregates or defects in signal transduction, which can lead to tissue injury and disease.

Farnesoid X Receptor (FXR) is a ligand-activated transcription factor and a member of the nuclear receptor superfamily. FXR is highly expressed in liver and intestine [12]. FXR can be activated by bile acids, which are its endogenous ligands [13]–[15], or by synthetic ligands such as GW4064 [16]. FXR activation is essential in maintaining bile-acid homeostasis via transcriptional regulation of nuclear receptors, bile-acid transporters, and the hormonal fibroblast growth factor Fgf15/19 [17].

In addition to its function in maintaining bile-acid homeostasis, FXR regulates lipid metabolism [18], [19], cholesterol metabolism [20], [21], liver regeneration [22], and glucose metabolism [23]. Recent studies also indicate a role for FXR in regulating innate immunity and inflammation [24]–[27]. Although the exact role of FXR in regulating tissue homeostasis is not clear, FXR deficiency leads to development of various disease states such as hepatocellular carcinoma [28], [29], intestinal tumorigenesis [30]–[32], intestinal inflammation [24], [26], [33], cholestasis [34], nonalcoholic steatohepatitis (NASH) [35] and gall stone formation [36].

A possible new role for FXR has been revealed through our discovery of a novel binding site within the *Sqstm1* gene in liver and ileum by genome-wide analysis [37]. However, it is unknown whether FXR can functionally regulate expression of the *Sqstm1* gene. If this hypothesis is verified, it may represent a mechanism by which FXR maintains tissue homeostasis and regulates inflammation. Therefore, the purpose of this study was to determine if binding of FXR to the *Sqstm1* gene in the liver and ileum produces a functional binding site capable of inducing transcriptional activation of the *Sqstm1* gene. Our findings indicate that FXR binds to the *Sqstm1* gene in both liver and ileum. However, activation of FXR only induces *Sqstm1* expression in the ileum but not in the liver, suggesting complex regulation of *Sqstm1* gene transcription in a tissue-specific manner. In addition, FXR-mediated induction of p62 may be a potential protective mechanism of FXR.

**Figure 1:**
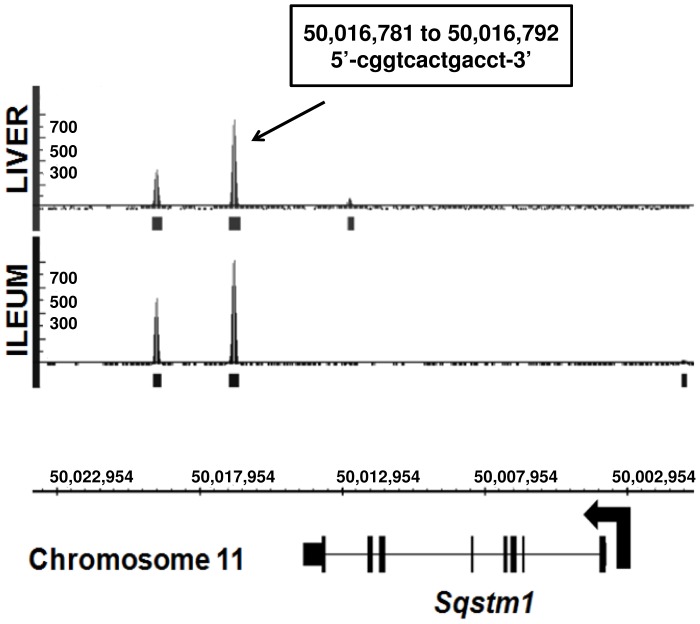
ChIP-sequencing results for FXR binding to *Sqstm1*. 10-week old WT mice were fasted overnight and then given a one-time treatment of vehicle or GW4064 (75 mg/kg) for four hours (liver) or two hours (ileum). Cross-linked sonicated genomic DNA extracted from livers and ileums were immunoprecipitated with antibody against FXR. ChIP-seq analysis revealed that FXR binds to two locations within the 3′ end of the *Sqstm1* gene in the liver and ileum as shown by two binding peaks located 13.1 kb and 15.8 kb downstream from the TSS of *Sqstm1* on chromosome 11. These peaks represent binding abundance. The highest binding peak in the intestine is represented by a peak value of 815, and the highest binding peak in the liver is represented by a peak value of 755. The 13.1 kb site contains a classical IR1, and the 15.8 kb site does not. The location within chromosome 11 is indicated for the IR1 at the 13.1 kb binding site along with the IR1 sequence. N = 3–4 mouse livers or ileums per group.

## Materials and Methods

### Animals and Treatment

Animals for Chromatin Immunoprecipitation (ChIP) studies were treated as previously described [37]. Briefly, 10-week old FXR knockout (FXR−/−) and wild-type (WT) mice with a C57BL/6 background were fasted overnight and then given a one-time treatment of vehicle (PBS with 1%Tween-20 and 1% methylcellulose) or GW4064 (75 mg/kg) by oral gavage for four hours before harvesting of their livers or two hours before harvesting of their ileums for ChIP-Seq analysis. For mRNA and protein level studies, ten to twelve-week old FXR−/− and WT mice were fasted overnight and received a one-time treatment of GW4064 (150 mg/kg) or vehicle by oral gavage for either 4 or 16 hours before harvesting of their livers and ileums for RNA and protein extraction. The VP-FXR transgenic mice were generated as previously described [38]. Briefly, constitutively active FXR was overexpressed in the liver and intestine using the tetracycline-inducible transgenic system. VP-FXR was generated by fusing the VP-16 transactivation domain from the herpes simplex virus to the 5′ end of the FXR cDNA. FXR−/− mice were generated as previously described [34]. All animal protocols were approved by the University of Kansas Medical Center Animal Care and Use Committee (protocol number 2010-1947), and the mice were cared for according to standard guidance. All efforts were made to minimize suffering.

**Figure 2:**
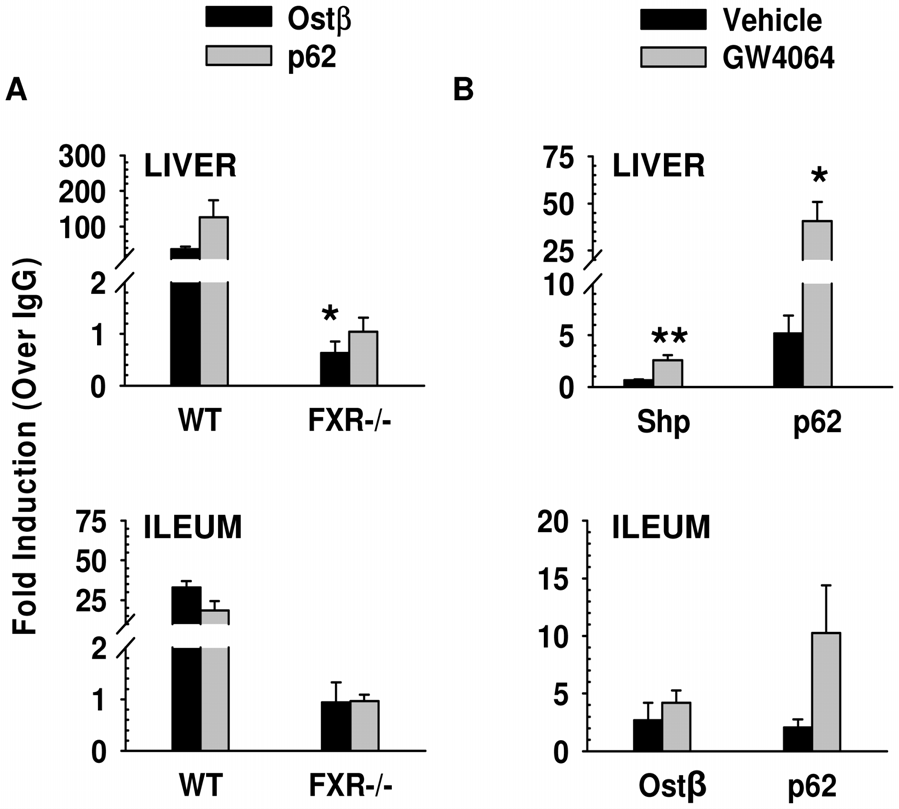
ChIP-qPCR results to confirm FXR binding to *Sqstm1*/p62 in liver and ileum. 10 to 12-week old FXR−/− and WT mice were fasted overnight and then given a one-time treatment of vehicle or GW4064 (75 mg/kg) for four hours (liver) or two hours (ileum). ChIP assay was performed using an antibody against FXR, and immunoprecipitated DNA was analyzed by qPCR. A: Vehicle-treated WT mice showed binding of FXR to *Sqstm1*/p62 in both liver (126-fold) and ileum (18-fold) when compared to IgG controls. This binding was reduced to IgG control levels in liver and ileum for vehicle-treated FXR −/− mice. *Ostβ* was used as a positive control for FXR binding in both liver and ileum. In the liver, there was a 36-fold increase in binding to *Ostβ* compared to IgG controls, and there was a 32-fold increase for binding to *Ostβ* in the ileum compared to IgG controls. This binding was reduced to IgG control levels in liver and ileum of vehicle-control treated FXR −/− mice (*indicates p<0.05, N = 3 WT and 4 FXR−/− mouse livers or ileums). B: Treatment of WT mice with the FXR agonist GW4064 increased FXR binding to the *Sqstm1*/p62 gene in both mouse liver and ileum. In liver, there was a 40-fold increase in FXR binding to the *Sqstm1*/p62 gene. The FXR target gene *Nr0b2*/Shp was used as a positive control for liver and showed a 2.5-fold increase after GW4064 treatment. In ileum, there was a 10-fold increase in FXR binding to *Sqstm1*/p62 with GW4064 treatment. The FXR target gene *Ostβ* was used as a positive control for ileum and showed a 4-fold increase after GW4064 treatment (*indicates p<0.05, **indicates p<0.01, N = 4 mouse livers or ileums per group).

### ChIP-Seq

Chromatin immunoprecipitation (ChIP) followed by massive parallel sequencing (ChIP-seq) analysis was performed as previously reported [37]. Briefly, cross-linked sonicated genomic DNA extracted from ten week-old fasted WT and FXR−/− male mouse livers or ileums gavaged with vehicle or GW4064 for 2 hours (ileum) or 4 hours (liver) were immunoprecipitated with antibody against FXR. Immunoprecipitated DNA fragments were then prepared for massive parallel sequencing analysis as previously described [37]. Enriched intervals, referred to as peak values, were identified when a given genomic region containing more than one enriched interval overlapping by at least one base pair appeared more than 20 times. Histograms of FXR binding to the *Sqstm1* gene in liver and ileum were generated by loading sequencing BAR files into Affymetrix Integrated Genome Browser (IGB) [39].

**Figure 3:**
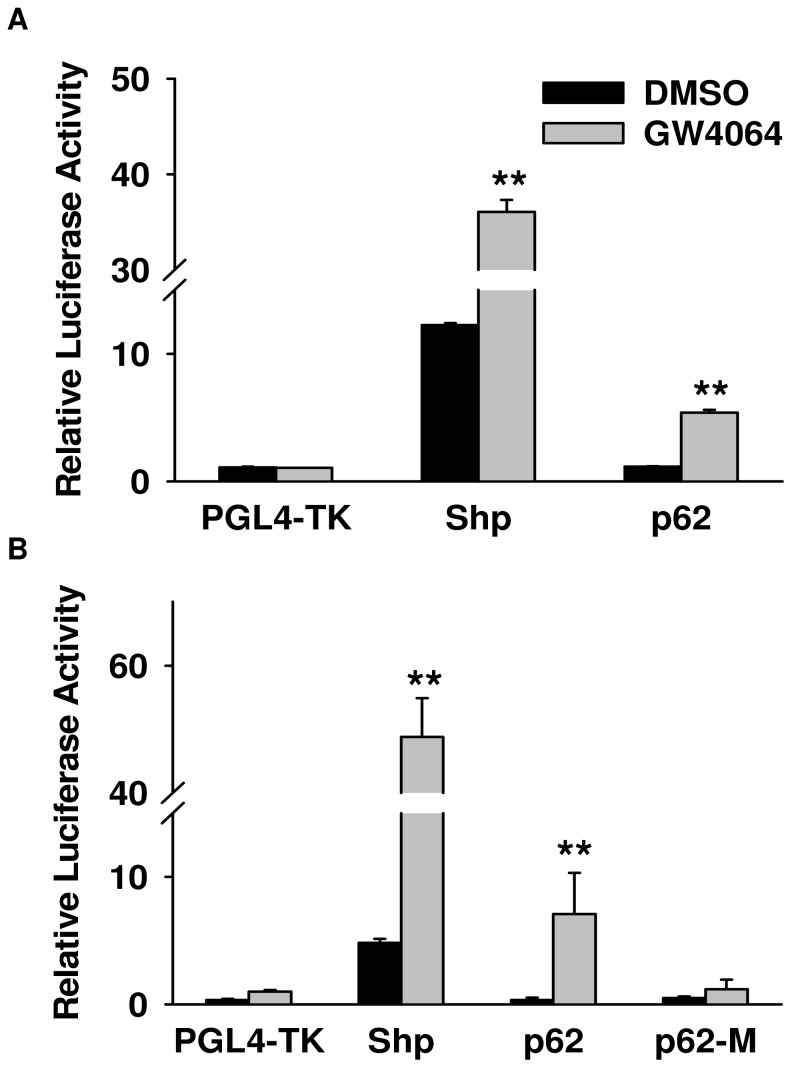
Activation of FXR enhanced *Sqstm1*/p62 transcriptional activation as revealed by luciferase assay. HepG2 cells were transfected with plasmid DNA containing either the PGL4-Shp-TK plasmid as a positive control or the plasmid DNA containing the 2 kb fragment of *Sqstm1*/p62 with the FXR IR1 (A) or the mutant IR1 (p62-M) (B). These plasmids were transfected along with human FXR, human RXRα and PCMV-renilla luciferase vector before the cells were treated with 1 µM GW4064 or 0.1% DMSO control for 36 to 48 hours. Firefly luciferase activity of each well was normalized as a ratio to that of renilla luciferase and expressed as fold over PGL4-TK empty vector control. The FXR target gene Shp was used as a positive control (**indicates p<0.01, N = 6 wells per treatment).

### ChIP-quantitative PCR (ChIP-qPCR)

ChIP was performed as previously described [37]. Briefly, ChIP assay was performed using anti-FXR antibody (H-130, Santa Cruz, CA), and immunoprecipitated DNA was analyzed by quantitative PCR (qPCR) using SYBR Green chemistry (Fermentas, Glen Burnie, Maryland). QPCR was performed to amplify FXR binding sites located in the *Nr0b2* and *Ostβ* genes, which are positive control regions for FXR binding, as well as for the novel FXR binding site in the *Sqstm1* gene. A novel FXR binding site identified by ChIP-seq analysis was located 13.1 kb downstream of the *Sqstm1* transcription start site (TSS). This site was amplified by ChIP qPCR analysis using primers: *Nr0b2* 3′ binding site F: 5′-CAGTCCACGCCCTCAGCCC-3′ and R: 5′-GGCAGGAGGAGGTCTGAAAGC-3′, *Ostβ* F: 5′-CCGCAATGGCAGATCATAC-3′ and R: 5′-GTGAATGACCCCACGAATG-3′, and *Sqstm1* F: 5′-CACTGCACATGTGTGTTTCTGTGT-3′ and R: 5′-AGGGTGTGGACAGTGTTGAAGACA-3′. ChIP-qPCR results were normalized to input and expressed as fold over IgG negative controls.

**Figure 4:**
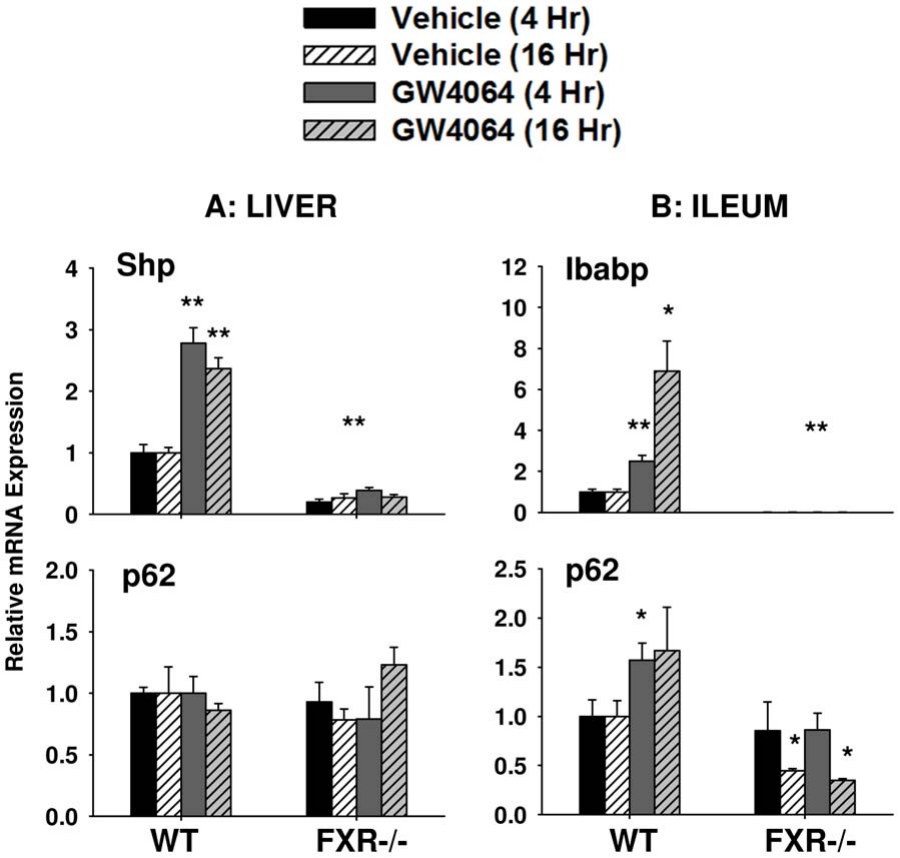
Relative mRNA expression levels of *Sqstm1*/p62 determined by quantitative PCR in WT and FXR−/− mouse liver (A) and ileum (B) after treatment with GW4064 for 4 or 16 hours. Ten to twelve week-old WT and FXR−/− mice were fasted overnight and received a one-time treatment of GW4064 (150 mg/kg) or vehicle for either 4 or 16 hours before removal of their livers and ileums for RNA isolation. *Sqstm1*/p62 mRNA expression was induced in WT ileum upon FXR activation with GW4064 but not in liver. In addition, basal expression levels of *Sqstm1*/p62 decreased in FXR−/− mice in ileum but not in liver. FXR target genes Shp and Ibabp were used as positive controls for liver and ileum, respectively. Expression of Shp and Ibabp was induced by GW4064 treatment in WT mice, and basal expression of these target genes decreased in FXR−/− mice as expected. Real-time qPCR results were normalized to 18 s and expressed as fold over WT vehicle control (*indicates p<0.05 and **indicates p<0.01, N = 5 for WT and N = 4 for FXR−/− mouse livers or ileums).

### RNA Isolation and Real-Time qPCR

RNA was isolated using TRI Reagent (Ambion, Applied Biosystems, Austin, TX) according to the manufacturer’s instructions, and RNA concentration was determined by spectrophotometry. cDNA was generated using standard RT-PCR protocols, and qPCR was performed using SYBR Green chemistry. The following primers were used for Real-Time qPCR: Shp F: 5′-CGATCCTCTTCAACCCAGATG-3′ and R: 5′-AGGGCTCCAAGCATTCACACA-3′, Ibabp F: 5′-GGTCTTCCAGGAGACGTGAT-3′ and R: 5′-ACATTCTTTGCCAATGGTGA-3′, and *Sqstm1/*p62 F: 5'-AGAATGTGGGGAGAGTGTG-3′ and R: 5'-TCGTCTCCTCCTGAGCAGTT-3′. Real-time qPCR results were normalized to 18 s and expressed as fold over WT vehicle control.

**Figure 5:**
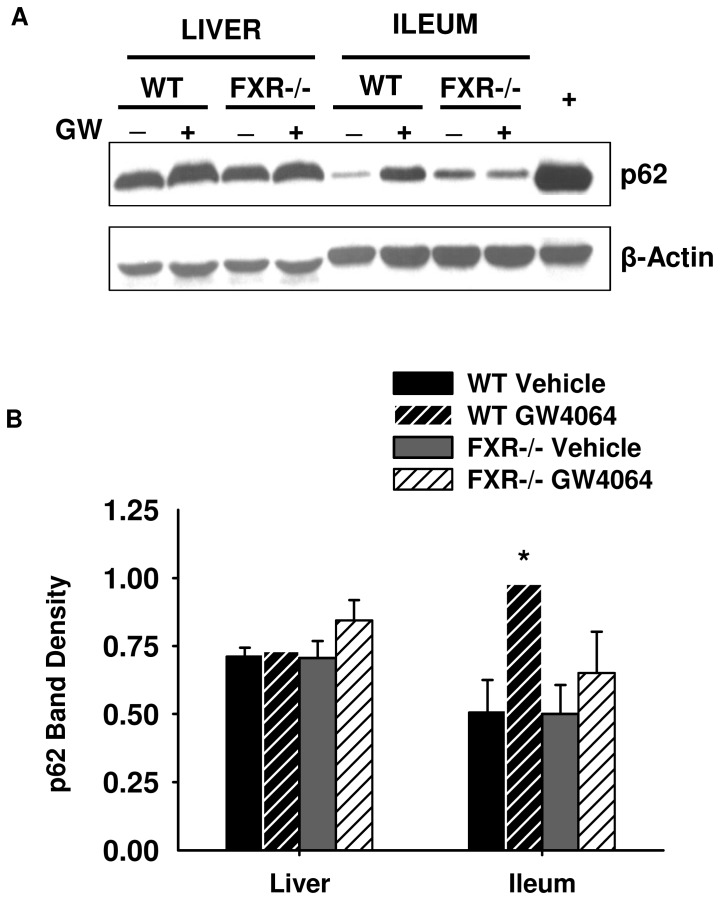
Western blot for p62 in mouse liver and ileum (A) with band density normalized to β-actin (B). WT and FXR−/− mice were treated with GW4064 (150 mg/kg) or vehicle for 16 hours before removal of their livers and ileums. Cytosolic extracts were used to determine p62 protein expression by western blot (A), and band density values were normalized to β-Actin (B). Protein expression of p62 increased in ileum upon FXR activation, but activation of FXR had no effect on p62 protein expression in the liver. The samples used for the blot shown are pooled liver and ileum samples from three mice. The error bars in the graph indicate results from the individual mouse livers and ileums run on separate blots, which are not shown. Band density was determined using ImageJ software (*indicates p<0.05, N = 3 mouse livers or ileums, + represents chloroquine treatment, which is a positive control for p62 expression).

### Construction of Plasmids

A 2 kb region of the *Sqstm1*/p62 gene containing a FXR response element, which is an inverted repeat separated by one nucleotide (IR1), was cloned into a PGL4-TK luciferase vector. This IR1 was located 13.1 kb downstream of the *Sqstm1*/p62 gene TSS. The cloned construct was confirmed by DNA sequencing, and the new plasmid was named PGL4-p62-TK luciferase vector.

**Figure 6:**
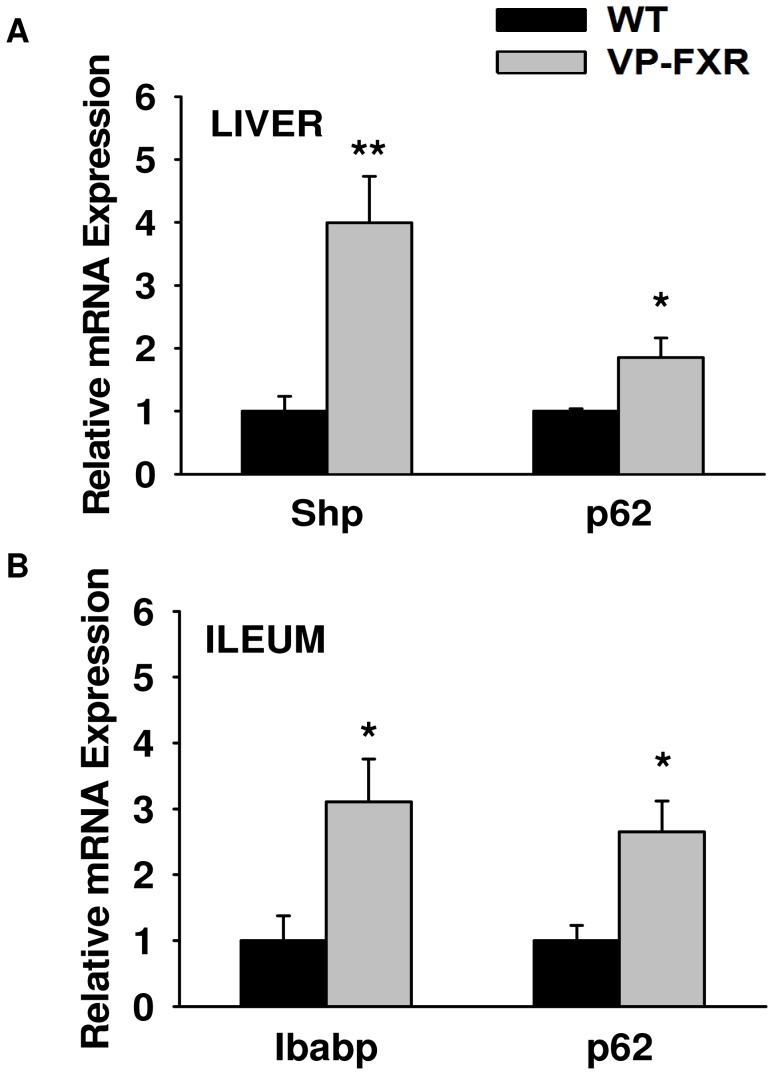
p62 mRNA expression in VP-FXR transgenic mouse liver (A) and ileum (B). Expression of *Sqstm1*/p62 mRNA was determined in VP-FXR transgenic mouse livers and ileums. Ibabp and Shp were used as positive controls for ileum and liver, respectively. Expression of *Sqstm1*/p62 mRNA was induced in both VP-FXR transgenic mouse liver and ileum compared to WT controls. Expression of positive control Shp and Ibabp mRNA was induced in transgenic mouse liver and ileum, respectively, when compared to WT controls (*indicates p<0.05, **indicates p<0.01, N = 5 mouse livers or ileums).

This IR1 was mutated in the PGL4-p62-TK vector using a QuikChange II XL Site-Directed Mutagenesis Kit (Stratagene, La Jolla, CA) according to the manufacturer’s instructions utilizing the following primers: F:5′-GCAATCCTACGTTGGCCCCAAGTTCACTGATGTGGTGTTCAAAGTTGTC-3′ and R: 5′-GACAACTTTGAACACCACATCAGTGAACTTGGGGCCAACGTAGGATTGC-3′. These primers generated an IR1 mutant by changing the IR1 sequence 5′-CGGTCACTGACCT-3′ to the mutant sequence 5′-AGTTCACTGATGT-3′. The mutated base pairs are underlined in the original sequence. The mutation was confirmed by DNA sequencing, and the mutated plasmid was named p62-M.

### Cell Culture, Transient Transfection, and Luciferase Reporter Gene Assay

HepG2 cells, purchased from the American Type Culture Collection (Manassas, VA), were cultured in DMEM supplemented with 10% fetal bovine serum (Omega Scientific, Tarzana, CA) and 1% penicillin/streptomycin in a 5% CO_2_ humidified atmosphere at 37°C. Cells were plated at a density of 5,000 cells per well in 200 µL medium in a 96-well plate and incubated overnight. Transient transfection was performed as previously described [40]. Briefly, cells were transfected with 0.2 µg plasmid per well containing either PGL4-p62-TK or p62-M along with human FXR, human RXRα and PCMV-renilla luciferase vector (Promega, Madison, WI) using TurboFect *in vitro* transfection reagent (Fermentas, Glen Burnie, Maryland) according to the manufacturer’s instructions. The previously described PGL4-Shp-TK plasmid [40] was used as a positive control for FXR activation. Six hours after transfection, medium was changed and cells were treated with 1 µM GW4064 or 0.1% DMSO as a control. Thirty six to forty eight hours later, firefly and renilla luciferase activities were measured using a Dual-Glo Luciferase Assay kit (Promega, Madison, WI). Firefly luciferase activity of each well was normalized as a ratio to that of renilla luciferase and expressed as fold over PGL4-TK empty vector control.

### Western Blot

Cytoplasmic extracts from FXR −/− and WT mouse liver and ileum were isolated using a NE-PER kit (Thermo Scientific, Fremont, CA) according to the manufacturer’s instructions. Protein concentration was measured using BCA assay (Thermo Scientific, Fremont, CA). Western Blot was performed using 20 µg of protein separated on a 10% SDS/PAGE gel and transferred to a 0.45 µm PVDF membrane (Millipore, Billerica, MA). The membrane was blocked with 5% non-fat milk in TBS before adding p62 antibody (1∶1000, Abnova, Walnut, CA). A chloroquine-treated HeLa cell lysate sample was used as a positive control for p62 labeling (molecular weight 62 kDa), and β-Actin (molecular weight 42 kDa) was used as a loading control. Band density was determined using ImageJ software.

### Statistics

A student’s *t*-test was used to determine statistical significance for samples that demonstrated equal variance. A Mann-Whitney Rank Sum test was used to determine statistical significance for samples that did not demonstrate equal variance. A p-value of <0.05 was considered statistically significant. A p-value of <0.05 is indicated by * and a p-value of <0.01 is indicated by **.

## Results

### FXR Binding to the *Sqstm1* Gene in Mouse Liver and Ileum

Binding of FXR to two regions at the 3′ end of the *Sqstm1* gene in the liver and ileum was discovered by our genome-wide ChIP-seq analysis [37]. These two FXR binding sites were located 13.1 and 15.8 kb downstream of the *Sqstm1* TSS on chromosome 11. Abundance of FXR binding to novel and known target genes in the liver and ileum in ChIP-seq results were interpreted by a binding peak value. The peak value of FXR binding to the *Sqstm1* gene at the 13.1 kb site was 755 in liver and 815 in ileum (Figure 1). The peak value of FXR binding to the *Sqstm1* gene at the 15.8 kb site was 330 in liver and 500 in ileum (Figure 1). Binding of FXR to the 13.1 kb site of the *Sqstm1* gene represented one of the highest peak values detected by ChIP-seq analysis, and it is relatively high compared to FXR binding to other known FXR target genes. For example, the peak value of FXR binding to the *Nr0b2* gene encoding small heterodimer partner (Shp) was 498, and the peak value of FXR binding to the *Ostβ* gene encoding organic solute transporter β (Ostβ) was 572 [37]. Furthermore, sequence analysis of the 13.1 kb FXR binding site within the *Sqstm1* gene by NUBIScan [41] revealed the presence of a classical IR1. The chromosomal location of this IR1 and its sequence are shown in Figure 1. The 15.8 kb binding site did not have an IR1 present according to NUBIScan [41]. Therefore, the 13.1 kb site was further analyzed as a functional FXR binding site.

The binding results from ChIP-seq were confirmed by ChIP-qPCR as shown in Figure 2. Vehicle-treated WT mice showed binding of FXR to *Sqstm1*/p62 in both liver (126-fold) and ileum (18-fold) when compared to IgG controls. This binding was reduced to IgG control levels in liver and ileum for vehicle-treated FXR −/− mice (Figure 2A). *Ostβ* is a known FXR target gene and was used as a positive control for FXR binding in both liver and ileum with a 36-fold and a 32-fold increase in binding in the liver and ileum, respectively, compared to IgG controls (Figure 2A). This binding was also reduced to IgG control levels in liver and ileum of vehicle-control treated FXR −/− mice (Figure 2A). Furthermore, treatment of WT mice with a FXR synthetic agonist, GW4064, increased FXR binding to the *Sqstm1* gene in both mouse liver and ileum (Figure 2B). In liver, there was an increase in FXR binding to the *Sqstm1*/p62 gene (40-fold, p<0.05) and to the *Nr0b2*/Shp gene (2.5 fold, p<0.01) with GW4064 treatment for 4 hours. In ileum, 2-hour GW4064 treatment resulted in an increase in FXR binding to both *Sqstm1*/p62 and *Ostβ* genes (10-fold and 4-fold, respectively).

### Activation of FXR Enhances Transcriptional Activation of *Sqstm1 as* Revealed by the Luciferase Reporter Gene Assay

A luciferase reporter assay was performed to determine if FXR binding to the *Sqstm1*/p62 gene was functional in enhancing transcription. Activation of FXR by GW4064 increased the luciferase activity of p62 3 to 7 fold (p<0.01, Figures 3A and 3B) when driven by an IR1 FXR response element (5′-CGGTCACTGACCT-3′) found 13.1 kb downstream of the *Sqstm1*/p62 gene TSS compared to PGL4-TK vector control (Figure 3A). In addition, mutation of this FXR response element (p62-M, 5′-AGTTCACTGATGT-3′) reduced luciferase activity to levels similar to the PGL4-TK vector control (Figure 3B). As a positive control, activation of FXR by GW4064 significantly enhanced luciferase activity approximately 3 to 4 fold (p<0.01) when driven by a FXR response element identified in the *Nr0b2*/Shp gene regulatory region (Figures 3A and 3B).

### Activation of FXR Induces mRNA Expression of *Sqstm1* in Ileum but not in Liver

Binding of FXR to the *Sqstm1* gene does not guarantee activation of the gene’s transcription because many factors are involved in gene transcriptional activation. Therefore, *Sqstm1*/p62 mRNA expression levels were determined following FXR activation using *Nr0b2* or *Fabp6* as positive controls. *Fabp6* is the gene encoding for ileum bile acid binding protein (Ibabp). Shp is a classical target gene of FXR in the liver, and Ibabp is a direct target gene of FXR in the ileum. A significant GW4064-mediated induction of Shp mRNA was observed in both the 4- (2.8-fold) and 16-hour (2.4-fold) treatment groups for WT mouse livers (P<0.01), as shown in Figure 4A. However, no induction of *Sqstm1*/p62 mRNA was seen for either time point in WT mouse livers (Figure 4A).

In contrast to results seen in the liver, a GW4064-mediated induction of both Ibabp and *Sqstm1*/p62 mRNA was observed in the 4- and 16-hour GW4064 treatment groups for WT mouse ileum when compared to vehicle controls (Figure 4B). Treatment with GW4064 resulted in a significant 2.5 and 6.9-fold induction of Ibabp (p<0.05) and a 1.6 and 1.7-fold induction in *Sqstm1*/p62 mRNA in the 4 and 16-hour treatment groups in WT mouse ileum, respectively. Only the 4-hour GW4064 treatment group induction of *Sqstm1*/p62 mRNA was statistically significant (p<0.05).

We then used FXR−/− mice to confirm that the GW4064-mediated induction of *Sqstm1* gene expression was due to FXR activation. As shown in Figure 4A, a significant decrease in baseline Shp expression levels in liver was seen in both vehicle- and GW4064-treated FXR −/− mice for the 4- and 16-hour treatment groups when compared to WT vehicle controls (p<0.01). However, FXR deficiency did not seem to affect *Sqstm1*/p62 baseline expression in mouse livers. For mouse ileums, a significant decrease in baseline Ibabp expression was seen in both vehicle- and GW4064-treated FXR −/− mice in the 4- and 16-hour treatment groups when compared to WT vehicle controls (p<0.01, Figure 4B). In addition, FXR −/− mice showed a baseline decrease in *Sqstm1*/p62 expression in ileum in both 4- and 16-hour GW4064 treatment groups, but this finding was only statistically significant for the 16-hour treatment group (p<0.05, Figure 4B).

### Protein Expression of p62 in Mouse Liver and Ileum

It is known that p62 is expressed in intestinal epithelia [42]. After we observed induction of *Sqstm1*/p62 mRNA by FXR activation in ileum, we determined whether increased mRNA levels translated into protein induction. As shown in Figure 5, GW4064 treatment significantly increased p62 protein expression 2-fold (p<0.05) over vehicle controls in mouse ileum. Furthermore, the GW4064-mediated induction of p62 protein expression was abolished in FXR−/− mouse ileums. However, there was no effect of GW4064 treatment on p62 protein expression in mouse liver.

### mRNA Expression of *Sqstm1* in FXR Transgenic Mouse Liver and Ileum

We used VP-FXR transgenic mice to determine whether genetically constitutive activation of FXR could also regulate the expression of *Sqstm1*/p62. As shown in Figure 6, *Sqstm1*/p62 mRNA expression was significantly increased in both liver and ileum from VP-FXR transgenic mice when compared to WT controls (p<0.05). Shp and Ibabp were used as positive controls and were also significantly increased in VP-FXR transgenic mouse liver and ileum, respectively, when compared to WT mice (p<0.05).

## Discussion

p62 is the protein encoded by the *Sqstm1* gene and has important cellular functions. In addition to the well-known role of p62 in facilitating selective autophagy, p62 also activates NF- κB [5]–[7], which is well known for its regulation of genes needed to promote cell survival and inflammation. In addition, p62 activates apoptosis to promote cell death [4] and activates the Nrf2 pathway to respond to oxidative stress [9]–[11].

FXR is a nuclear receptor suspected to have a role in the regulation of homeostasis in both liver and intestine. In the current study, we revealed that *Sqstm1* is a *bona fide* FXR target gene by showing a novel FXR binding site located within the *Sqstm1* gene in mouse liver and ileum using ChIP-seq analysis (Figure 1). In addition, treatment with the synthetic ligand of FXR, GW4064, increased binding of FXR to this novel target gene in both liver and ileum, and this binding was significantly reduced in FXR−/− mice (Figure 2). Furthermore, binding of FXR to the *Sqstm1* gene regulatory region led to increased transcriptional activation as confirmed by luciferase reporter assay, and this transcriptional activation was abolished when the IR1 FXR response element was mutated (Figure 3). However, it appears that induction of *Sqstm1* is tissue-specific because mice treated with GW4064 to activate FXR only had an induced expression of *Sqstm1* in the ileum but not in the liver (Figure 4). The results from this study provide a potential mechanism by which FXR regulates the inflammatory response and/or promotes cellular homeostasis by inducing transcription of p62.

The tissue-specific induction of *Sqstm*1/p62 mRNA, despite the fact that FXR binds to a gene regulatory region of *Sqstm1* in both liver and ileum, is an intriguing observation. This suggests that p62 is regulated by multiple transcription factors. For example, another known transcriptional regulator of p62 is Nrf2, which is important for regulating the oxidative stress response [9]. Therefore, the presence and/or balance of these various transcription factors, and possible inhibitory factors, that regulate p62 expression may determine whether FXR binding will be translated into transcriptional activation of the *Sqstm1* gene. In addition, the fact that *Sqstm1*/p62 expression is not induced in the liver with FXR activation could be due to higher basal expression of *Sqstm1*/p62 in the liver than in the ileum.

Even though induction of *Sqstm1* mRNA expression and p62 protein expression was only seen in mouse ileum and not in mouse liver after FXR activation (Figures 4 and 5), there was an increase in *Sqstm1* mRNA expression in both the liver and ileum of the VP-FXR transgenic mice (Figure 6). This increase in *Sqstm1* mRNA expression in the liver of VP-FXR transgenic mice could be due to the presence of constitutively active FXR, which is not present in WT mice treated with GW4064 to activate FXR. Assuming the inability of FXR activation to induce *Sqstm1* expression in the liver is due to the presence of a co-repressor or inhibitory transcription factor, then constitutively active FXR in the transgenic mice might be strong enough to remove or override a competing transcription factor or co-repressor bound to the *Sqstm1* promoter, and therefore, promotes transcription of the gene.

The specific IR1 sequence found in mouse is not conserved in human. However, there are several IR1 sites located within this downstream region on Chromosome 5 in human according to analysis by NUBIScan [41]. FXR may regulate *Sqstm1* gene expression in human by binding to one of these IR1 response elements. Furthermore, the ileum-specific regulation of *Sqstm1/*p62 by FXR may have an implication in intestinal diseases. FXR deficiency has been shown to cause intestinal injury and disease such as inflammation [24], [26], [33] and tumorigenesis [30]–[32]. In addition, FXR has been shown to play a role in maintaining intestinal epithelial cell proliferation to protect against tumorigenesis [31], [32]. Therefore, it is possible that FXR regulates the expression of the *Sqstm1* gene in the ileum in order to mediate selective autophagy or signal transduction to maintain cellular homeostasis, regulate the inflammatory response, and/or conduct tissue repair. If FXR is indeed regulating *Sqstm1*/p62 expression for these processes, then tissue-specific drug development of a synthetic activator of FXR in the intestine could be beneficial for treating or preventing intestinal diseases. This tissue-specific role of FXR in *Sqstm1* gene regulation is a novel finding and subsequent studies will further investigate the role of FXR in the regulation of *Sqstm1*/p62.

In conclusion, it is known that both p62 and FXR have beneficial effects in maintaining cellular homeostasis and preventing disease. We have shown that FXR transcriptionally regulates p62 expression in the intestine. Understanding the role of FXR’s regulation of p62 may further advance our understanding of p62 function, as well as the underlying molecular mechanism of FXR targeted pathways.
